# The Use of Ultrasound as a Potential Adjunct to Cell-Free Fetal DNA Screening for Aneuploidy at Weill Cornell Medical College, New York, USA

**DOI:** 10.1055/s-0038-1624564

**Published:** 2018-02-09

**Authors:** Jessica Scholl, Stephen Chasen

**Affiliations:** 1 Department of Obstetrics and Gynecology, Weill Cornell Medical College, New York, New York

**Keywords:** Cell-free fetal DNA, prenatal cytogenetics, fetal ultrasound, nuchal translucency

## Abstract

**Objective** To evaluate the utility of ultrasound in identifying fetuses with uncommon chromosomal abnormalities that would be considered not detectable by cell-free fetal deoxyribonucleic acid (cfDNA).

**Study Design** We performed a retrospective study of fetuses with chromosomal abnormalities that would be undetectable by cfDNA, who underwent an 11- to 14-week ultrasound from 2006 to 2016.

**Results** There were 43 pregnancies included. First-trimester ultrasound revealed a fetal abnormality in 19 (44.2%) cases, of which 13 (30.2%) had a thickened nuchal translucency. There were an additional four fetuses with second-trimester sonographic abnormalities. Overall, 23 (53.5%) fetuses were found to have a major anomaly diagnosed by ultrasound. The rate of first-trimester sonographic abnormalities varied widely based on category of chromosomal abnormalities with high rates seen with triploidy (87.5%) and autosomal trisomy (80%) and lower rates seen with structurally abnormal chromosomes (33.3%), trisomy mosaicism (27.3%), other forms of mosaicism (11.1%), and deletions or duplications (25.0%), *p* < 0.001.

**Conclusion** The majority of fetuses with uncommon chromosomal abnormalities in our cohort had major sonographic anomalies. The use of first-trimester ultrasound with nuchal translucency measurement may offer utility in identifying fetuses with risk of aneuploidy that would not be detectable with cfDNA.

The implementation of noninvasive prenatal screening using cell-free fetal deoxyribonucleic acid (cfDNA) and its rapid induction into clinical care have led to a significant shift in prenatal screening algorithms. 1 2 Prior to the introduction of cfDNA in 2011, conventional screening methods utilizing a combination of maternal serum analytes with measurement of the fetal nuchal translucency were the mainstay of fetal aneuploidy screening. Conventional methods using the integrated or sequential approach have been reported to detect 90 to 95% of Down syndrome cases with a false positive rate of 5%. 3 4 5 In contrast, cfDNA has been identified to have a higher sensitivity (>99%), lower false positive rate (0.15%), and higher positive predictive value than conventional screening for Down syndrome detection. 6 7 8 9 10 11 It has also demonstrated high sensitivity and specificity for trisomy 18, with somewhat lower sensitivity for trisomy 13 and sex chromosome abnormalities. 2 8 11 12 13 For these reasons, cfDNA is selected by many patients as a primary screening method for fetal aneuploidy.

Despite the high test performance for common chromosomal abnormalities, cfDNA does not detect non-targeted aneuploidies. 1 Indeed, a potential advantage to conventional screening is that patients who “screen positive” for trisomy 21 or trisomy 18/13 have been identified to have significant abnormalities including triploidy, rare trisomies, deletions or duplications, and mosaicisms that are considered undetectable by cfDNA. 14 15 16 17 It has been estimated that 17% of chromosomal abnormalities identified by conventional screening are considered not detectable by cfDNA, with sequelae ranging from mild conditions to significant disabilities. 1

While recognizing the benefits and limitations of each screening paradigm, the American Congress of Obstetricians and Gynecologists (ACOG) recommends against parallel or simultaneous testing with multiple screening modalities. 2 However, it remains unclear whether first-trimester ultrasound alone, without maternal serum analytes, is a useful adjunct in identifying uncommon fetal chromosomal abnormalities. An enlarged nuchal translucency, notably, was found among 19% of pregnancies that were “screen positive” with a cfDNA-undetectable abnormality. 1 Our objective was to evaluate the utility of ultrasound in identifying fetuses with chromosomal abnormalities, which would be considered not detectable by cfDNA.

## Materials and Methods

We performed a retrospective, cross-sectional, descriptive study of pregnancies with fetal chromosomal abnormalities, which would be considered undetectable by cfDNA tests. All patients had been seen in a single academic medical center during the period from January 2006 to March 2016. The study was approved by the Institutional Review Board at Weill Cornell Medical College. This medical center provides ultrasound, genetic counseling, and prenatal diagnosis services, as well as labor and delivery and neonatal intensive care services. All sonographers are non-physicians and perform nuchal translucency assessments according to established guidelines, while adhering to ongoing quality assurance by the Nuchal Translucency Quality Review Program (NTQR). 18 19 All nuchal translucency ultrasounds are read and interpreted by Maternal–Fetal Medicine attending physicians, with credentials by either NTQR or the Fetal Medicine Foundation. 18 20

Inclusion criteria were pregnancies from 2006 to 2016 with a fetus having a chromosomal abnormality that would be undetectable by cfDNA, and who underwent an 11- to 14-week ultrasound. Fetuses with abnormal karyotypes were identified using a common database in our prenatal diagnosis unit. This database includes all fetuses found to have an abnormal karyotype by invasive testing with amniocentesis or chorionic villus sampling (CVS) performed in our ultrasound unit, infants who were identified postnatally with an abnormal karyotype who underwent prenatal sonography in our unit, and pregnancies ending in termination with an abnormal karyotype found on tissue sampling. Over the 10-year study period, there were ∼275 pregnancies found to have an abnormal karyotype.

An abnormal karyotype was defined as a karyotype other than 46, XX or 46, XY by cells obtained from CVS, amniocentesis, or tissue sampling. Tissue sampling included products of conception following a termination of pregnancy as well as neonatal sampling. Abnormal karyotypes were then classified into whether they were detectable or not detectable by cfDNA screening tests. Non-mosaic trisomy 13, 18, or 21, or sex chromosome aneuploidy were considered detectable by cfDNA and were excluded from the study. Other rare trisomies, triploidy, structural rearrangements, including unbalanced translocations, duplications, and deletions, and all forms of mosacism were considered not detectable by cfDNA and were thus included in the cohort. 1 3 We did not include apparently balanced translocations that were not detectable by cfDNA because of their low risk of phenotypic effect.

All data were collected from the institutional electronic databases. The data were extracted using a standardized data collection form and entered into a standardized spreadsheet. Information obtained from hospital records included maternal age, race or ethnicity, and parity, as well as indication and method for invasive testing, and fetal karyotype. Sonographic data including singleton or multiple gestation, nuchal translucency thickness, fetal crown-rump length (CRL) at nuchal translucency measurement, gestational age, and fetal morphology in the first and second trimesters were obtained from ultrasound and echocardiogram reports.

The outcomes assessed were abnormal findings on either first- or second-trimester fetal ultrasound. First-trimester ultrasound abnormalities included thickened nuchal translucency measurements, structural anomalies, and abnormal fetal biometry. Thickened nuchal translucency was defined as a nuchal translucency measurement greater than the 95 ^th^ percentile for fetal CRL. 20 Second-trimester ultrasound abnormalities included major congenital anomalies, defined as structural malformations that would be life threatening, result in long-term disability, or negatively affect neonatal or pediatric outcomes. 21 Soft markers, such as echogenic intracardiac foci, pyelectasis, or choroid plexus cysts, were not included in this definition.

Statistical analysis was performed using the IBM Statistical Package for the Social Sciences (IBM SPSS version 21; IBM Corporation INC, Armonk, NY). Descriptive statistics were calculated including frequencies, means, standard deviations, medians, and interquartile ranges. Chi-square analysis was used for statistical comparison.

## Results

There were 46 pregnancies with fetal chromosomal abnormalities other than trisomies 13, 18, 21, or sex chromosomal aneuploidy, in which an 11- to 14- week ultrasound had been performed. Three pregnancies with abnormal karyotypes identified by CVS were considered to have confined placental mosaicism and were thus excluded from the cohort. Therefore, there were 43 pregnancies included in the analysis. Characteristics of the study cohort are presented in Table 1 .

**Table 1:** Characteristics of cohort with fetal chromosomal abnormalities considered undetectable by cfDNA

*N*	43
Median maternal age, *y* (IQR)	36.5, (34–40)
Multiparous, *N* (%)	22 (51.2)
Singleton, *N* (%)	42 (97.7)
Race or ethnicity, *N* (%)	
White non-Hispanic	21 (48.8)
Black non-Hispanic	3 (7.0)
Hispanic	6 (14.0)
Asian	4 (9.3)
Other/unknown	9 (20.9)
Diagnosis by invasive test, *N* (%)	
CVS	25 (58.1)
Amniocentesis	13 (30.2)
Tissue sampling	5 (11.6)
Indications for testing, *N* (%)	
Abnormal first trimester screen	26 (60.5)
Advanced maternal age	6 (14)
Family history	3 (7.0)
Abnormal anatomy ultrasound	2 (4.7)
Maternal concern	2 (4.7)
Abnormal cfDNA results	2 (4.7)
Abnormalities at birth	1 (2.3)
Abnormal sequential screen	1 (2.3)

Abbreviations: cfDNA, cell-free fetal DNA; CVS, chorionic villus sampling; IQR, interquartile range.

The distribution of fetal chromosomal abnormalities included in the cohort was as follows: 5 (11.6%) with autosomal trisomy, 11 (25.6%) with mosaic trisomy, 8 (18.6%) with triploidy, 9 (21%) with other forms of mosaicism, 3 (7%) with unbalanced translocations, 4 (9.3%) with deletions or duplications, and 3 (7%) other structurally abnormal chromosomes, which included ring chromosomes, marker chromosomes, and partial trisomy. Of the 20 fetuses with mosaic cytogenetic abnormalities, 10 were diagnosed by amniocentesis, 6 were diagnosed by CVS only, and 4 underwent CVS followed by amniocentesis to confirm true fetal mosaicism. Among the study cohort, there were two with abnormalities categorized as undetectable by cfDNA for the purposes of this study, that were actually identified with an abnormal cfDNA result. One involved an additional ring chromosome 18, and the other was a mosaicism of 4 different cell lines involving chromosome 13.

Of the 43 included pregnancies, the 11- to 14-week scan revealed a fetal abnormality in 19 (44.2%) cases. Thirteen (30.2%) fetuses had a thickened nuchal translucency, with median measurement of 3.5 mm and range of 2.4 mm to 6.7 mm (interquartile range: 2.7–5.5 mm). In nine cases, the thickened nuchal translucency was an isolated finding. In four cases, there were additional abnormalities including cardiac and bladder anomalies, a sacral mass, and abnormal growth. Overall, four (9.3%) fetuses had abnormal growth. This included those with more than a 7-day discrepancy between CRL and well-established dates by either an early first-trimester ultrasound or by assisted reproductive technology-derived dating. 22 Also included among fetuses with abnormal growth were those with ratios of head circumference to abdominal circumference (HC/AC) greater than the 95 ^th^ percentile, indicating large head measurements relative to body measurements. 23 One fetus was found to have multiple anomalies including micrognathia, bilateral club feet, and echogenic kidneys. One fetus had an abnormal appearing brain with micrognathia, and one fetus had a prominent bladder ( Table 2 ).

**Table 2:** Fetal chromosomal abnormalities with associated ultrasound findings

Chromosomal abnormality, *N*	Abnormal first trimester ultrasound, *N* (%)	Abnormal second trimester ultrasound findings
Autosomal trisomy, 5 – Trisomies 4, 9, 10, 15, and 16	4 (80) – Thickened NT – Thickened NT and cardiac anomaly – Prominent bladder – Abnormal brain and micrognathia	– Growth restriction, abnormal profile, midline facial cleft, cardiac anomaly
Mosaic trisomy, 11 – Mosaic trisomies 7, 8, 9, 16, 20, and 22	3 (27.3) – 3 thickened NT	– Early growth restriction – Congenital heart defect – Hypoplastic right heart
Triploidy, 8	7 (87.5) – 3 small CRL, large head relative to body – Small CRL, abnormal profile, thickened NT – Thickened NT – Thickened NT and sacral mass – Thickened NT and enlarged bladder	
Other Mosaicism, 9	1 (11.1) – Micrognathia, bilateral club feet, echogenic kidneys	– Congenital diaphragmatic hernia
Unbalanced translocations, 3	2 (66.7) – 2 thickened NT	– Abnormal genitalia, thickened nuchal fold, fetal skin edema
Deletions/duplications 4	1 (25) – Thickened NT	– Ventriculomegaly, spina bifida, orofacial cleft Dandy–Walker variant
Other structurally abnormal chromosomes, 3	1 (33.3) – Thickened NT	– Severe micrognathia, orofacial cleft, nasal abnormality

Abbreviations: CRL, crown-rump length; NT, nuchal translucency.

The rate of first-trimester sonographic abnormalities varied widely based on category of chromosomal abnormalities with high rates seen with triploidy (87.5%) and autosomal trisomy (80%) and lower rates seen with structurally abnormal chromosomes (33.3%), trisomy mosaicism (27.3%), other forms of mosaicism (11.1%), and deletions or duplications (25.0%), *p* < 0.001.

A second-trimester anatomic survey was available on 17 (39.5%) pregnancies, performed between 16 and 20 weeks of gestation. First-trimester abnormalities had been identified in five of these. There were 12 pregnancies with a normal first-trimester ultrasound and second-trimester anatomic data, of which 4 (33.3%) fetuses were found to have a major abnormality. These included early severe growth restriction, facial abnormalities, congenital diaphragmatic hernia, and Dandy–Walker variant ( Table 2 ).

Overall, of the 43 included pregnancies, there were 23 (53.5%) fetuses with a major abnormality diagnosed by ultrasound.

## Discussion

The majority of fetuses with uncommon chromosomal abnormalities, considered undetectable by cfDNA, were found to have major ultrasound abnormalities. In 44% of our cohort, an abnormality was discovered during the 11-to 14-week scan, with a large subset involving a thickened nuchal translucency measurement.

In our center, an academic medical center with a large referral population, there is a lack of consistency among providers as to how to incorporate cfDNA results, prenatal ultrasound, and measurement of maternal biochemistry into a prenatal screening algorithm. While cfDNA became clinically available as a screening option for women with increased risk of fetal aneuploidy in 2011, the large majority of patients at our institution continued to undergo first-trimester ultrasound with serum analyte screening, independent of whether they also had cfDNA testing.

Guidelines have been unclear as to whether to perform nuchal translucency imaging in patients who have undergone cfDNA screening. ACOG states that while a nuchal measurement for aneuploidy risk is not necessary, ultrasound is useful to confirm viability and number of fetuses, assign gestational age, and identify some major fetal anomalies. 5 The International Society of Ultrasound in Obstetrics and Gynecology (ISUOG) recommends that among women with a negative cfDNA test result, first-trimester ultrasound should be offered and nuchal translucency thickness should be measured and reported as a raw value and centile. However, computing the first-trimester risk assessments for trisomy 21, trisomy 18, and trisomy 13 based on both nuchal translucency measurements *and* maternal biochemistry is not necessary. 24 Furthermore, the Society of Maternal–Fetal Medicine has recently stated that in women who have had a negative cfDNA screen, first-trimester nuchal translucency screening may slightly reduce the residual risk of significant chromosomal abnormalities; however, further research is needed to determine the optimal approach. 25 While these statements provide some guidance, there continues to be large variability in actual practice.

Recent publications have also endorsed the need for first-trimester scan for early detection of major structural anomalies that would be missed if cfDNA were used as the primary method of prenatal screening. 26 Among patients with negative cfDNA screening who underwent first-trimester ultrasound with nuchal translucency measurement, Reiff et al reported an abnormal fetal finding in 2.1% that placed them at increased risk of other genetic and/or structural anomalies. 27 We would agree with the recommendation to perform first-trimester ultrasound with measurement of nuchal translucency in patients who undergo cfDNA screening. However, in accordance with ACOG and ISUOG guidelines, we would caution against measurement of maternal biochemical analytes at the time of nuchal translucency scan, which will result in high positive screening rates and will deliver confusing risk estimates to patients without significantly enhancing detection rates. 5

The strength of this study is the ability to focus on a well-defined cohort of fetuses with uncommon chromosomal anomalies, which allowed us to describe the ultrasound findings in this population. Norton et al found that while considering all chromosomal abnormalities, the detection rate of sequential screen was greater than that of cfDNA screening, and attributed this to the sequential screen's ability to detect rare chromosomal abnormalities. 3 Our study assessed the rare abnormalities that are considered undetectable by cfDNA and found that many would be identifiable by prenatal ultrasound. While our study was not designed to evaluate the test performance of a screening algorithm using cfDNA plus ultrasound, our results suggest that ultrasound may be useful in detecting additional chromosomal abnormalities missed by cfDNA.

Our study was limited by its retrospective design and small sample size. Fetuses were included if they were found to have an abnormal karyotype that was considered undetectable by cfDNA. Since not all pregnancies undergo invasive testing, there were, no doubt, chromosomal abnormalities that were undiagnosed. Therefore, included cases may not be representative of the entire population of fetuses with rare abnormal karyotypes. In addition, the potential for selection bias exists, as a subset of patients in the group underwent invasive testing due to an ultrasound abnormality itself.

Because of the retrospective nature of the study, the available second-trimester data were limited. Many patients did not undergo a detailed anatomy ultrasound because they chose to terminate their pregnancy in the first or early second trimester. Thus, the observed rate of second-trimester sonographic abnormalities in this study is likely to be an underestimate. In addition, not all patients with mosaicism diagnosed by CVS underwent a second-trimester amniocentesis to rule out confined placental mosaicism. Thus, the possibility exists that several fetuses with confined placental mosaicism and no ultrasound abnormalities were included in the cohort, and our results, therefore, may underestimate the rate of abnormal ultrasound findings.

We recognize that cfDNA technology continues to evolve and that its capability to identify genetic conditions other than the common trisomies will likely expand. Some laboratories performing cfDNA assessment may screen for conditions other than trisomy 21, 18, or 13, such as triploidy. As more of the genome is evaluated with cfDNA, the role of ultrasound may diminish. In our study, notably, two rare abnormalities that were categorized as “undetectable” by cfDNA for the purposes of this study were actually identified with cfDNA. While cfDNA has been observed to screen positive for some rare karyotypes, at the current time, screening for mosaicism, and other chromosomal abnormalities have not been validated in clinical studies, and the sensitivity and specificity of this screening test is uncertain. 2 26

In the age of cfDNA screening, many women hope to avoid invasive testing, yet desire comprehensive detection of a wide range of conditions. 28 While this is not yet possible, we do suggest that the use of ultrasound may offer utility in identifying fetuses with risk of rare aneuploidy, which would not be detectable by cfDNA. We advocate considering the continued use of the 11- to 14-week scan, which allows the opportunity for detection of anomalies, options for diagnostic testing, appropriate counseling, and earlier decision making. We acknowledge that no definitive deductions can be made based on this small sample size; however, we do look forward to future research to investigate this proposed screening algorithm and to derive measures of test performance when using ultrasound with cfDNA to screen for rare fetal chromosomal abnormalities.

## References

[1] Norton M E Jelliffe-Pawlowski L L Currier R J Chromosome abnormalities detected by current prenatal screening and noninvasive prenatal testing Obstet Gynecol 2014 124 05 979 986 25437727 10.1097/AOG.0000000000000452

[2] Committee Opinion No.Committee opinion no. 640: cell-free DNA screening for fetal aneuploidy Obstet Gynecol 2015 126 03 e31 e37 26287791 10.1097/AOG.0000000000001051

[3] Norton M E Baer R J Wapner R J Kuppermann M Jelliffe-Pawlowski L L Currier R J Cell-free DNA vs sequential screening for the detection of fetal chromosomal abnormalities Am J Obstet Gynecol 2016 214 06 7270 7.27E8 10.1016/j.ajog.2015.12.018 26709085

[4] Malone F D Canick J A Ball R H First-trimester or second-trimester screening, or both, for Down's syndrome N Engl J Med 2005 353 19 2001 2011 16282175 10.1056/NEJMoa043693

[5] Practice bulletin no. 163: screening for fetal aneuploidy Obstet Gynecol 2016 127 05 e123 e137 26938574 10.1097/AOG.0000000000001406

[6] Bianchi D W Parker R L Wentworth J DNA sequencing versus standard prenatal aneuploidy screening N Engl J Med 2014 370 09 799 808 24571752 10.1056/NEJMoa1311037

[7] Nicolaides K H Syngelaki A Ashoor G Birdir C Touzet G Noninvasive prenatal testing for fetal trisomies in a routinely screened first-trimester population Am J Obstet Gynecol 2012 207 05 3740 3.74E8 10.1016/j.ajog.2012.08.033 23107079

[8] Norton M E Jacobsson B Swamy G K Cell-free DNA analysis for noninvasive examination of trisomy N Engl J Med 2015 372 17 1589 1597 25830321 10.1056/NEJMoa1407349

[9] Ashoor G Syngelaki A Wagner M Birdir C Nicolaides K H Chromosome-selective sequencing of maternal plasma cell-free DNA for first-trimester detection of trisomy 21 and trisomy 18 Am J Obstet Gynecol 2012 206 04 3220 3.22E7 10.1016/j.ajog.2012.01.029 22464073

[10] Gil M M Quezada M S Bregant B Ferraro M Nicolaides K H Implementation of maternal blood cell-free DNA testing in early screening for aneuploidies Ultrasound Obstet Gynecol 2013 42 01 34 40 23744609 10.1002/uog.12504

[11] Norton M E Brar H Weiss J Non-Invasive Chromosomal Evaluation (NICE) Study: results of a multicenter prospective cohort study for detection of fetal trisomy 21 and trisomy 18 Am J Obstet Gynecol 2012 207 02 1370 1.37E10 10.1016/j.ajog.2012.05.021 22742782

[12] Zimmermann B Hill M Gemelos G Noninvasive prenatal aneuploidy testing of chromosomes 13, 18, 21, X, and Y, using targeted sequencing of polymorphic loci Prenat Diagn 2012 32 13 1233 1241 23108718 10.1002/pd.3993 PMC3548605

[13] Porreco R P Garite T J Maurel K Noninvasive prenatal screening for fetal trisomies 21, 18, 13 and the common sex chromosome aneuploidies from maternal blood using massively parallel genomic sequencing of DNA Am J Obstet Gynecol 2014 211 04 3650 3.65E14 10.1016/j.ajog.2014.03.042 24657131

[14] Syngelaki A Pergament E Homfray T Akolekar R Nicolaides K H Replacing the combined test by cell-free DNA testing in screening for trisomies 21, 18 and 13: impact on the diagnosis of other chromosomal abnormalities Fetal Diagn Ther 2014 35 03 174 184 24525399 10.1159/000358388

[15] Alamillo C M Krantz D Evans M Fiddler M Pergament E Nearly a third of abnormalities found after first-trimester screening are different than expected: 10-year experience from a single center Prenat Diagn 2013 33 03 251 256 23354915 10.1002/pd.4054

[16] Shani H Goldwaser T Keating J Klugman S Chromosomal abnormalities not currently detected by cell-free fetal DNA: a retrospective analysis at a single center Am J Obstet Gynecol 2016 214 06 7290 7.29E13 10.1016/j.ajog.2015.12.025 26721783

[17] Gregg A R Gross S J Best R G ACMG statement on noninvasive prenatal screening for fetal aneuploidy Genet Med 2013 15 05 395 398 23558255 10.1038/gim.2013.29

[18] Abuhamad A Technical aspects of nuchal translucency measurement Semin Perinatol 2005 29 06 376 379 16533650 10.1053/j.semperi.2005.12.004

[19] D'Alton M E Cleary-Goldman J Education and quality review for nuchal translucency ultrasound Semin Perinatol 2005 29 06 380 385 16533651 10.1053/j.semperi.2006.01.004

[20] Nicolaides K H First-trimester screening for chromosomal abnormalities Semin Perinatol 2005 29 04 190 194 16104667 10.1053/j.semperi.2005.06.001

[21] Executive Board of the World Health Organization.Birth Defects: Report by the Secretariat. 126th Session Geneva, Switzerland World Health Organization 2009

[22] Committee opinion no. 700: methods for estimating the due date Obstet Gynecol 2017 129 05 e150 e154 28426621 10.1097/AOG.0000000000002046

[23] von Kaisenberg C S Fritzer E Kühling H Jonat W Fetal transabdominal biometry at 11-14 weeks of gestation Ultrasound Obstet Gynecol 2002 20 06 564 574 12493045 10.1046/j.1469-0705.2002.00842.x

[24] Salomon L J Alfirevic Z Audibert F ISUOG updated consensus statement on the impact of cfDNA aneuploidy testing on screening policies and prenatal ultrasound practice Ultrasound Obstet Gynecol 2017 49 06 815 816 28573775 10.1002/uog.17483

[25] Norton M E Biggio J R Kuller J A Blackwell S C The role of ultrasound in women who undergo cell-free DNA screening Am J Obstet Gynecol 2017 216 03 B2 B7 28108156 10.1016/j.ajog.2017.01.005

[26] Rao R R Valderramos S G Silverman N S Han C S Platt L D The value of the first trimester ultrasound in the era of cell free DNA screening Prenat Diagn 2016 36 13 1192 1198 27813114 10.1002/pd.4955

[27] Reiff E S Little S E Dobson L Wilkins-Haug L Bromley B What is the role of the 11- to 14-week ultrasound in women with negative cell-free DNA screening for aneuploidy? Prenat Diagn 2016 36 03 260 265 26748490 10.1002/pd.4774

[28] Norton M E Kuppermann M Women should decide which conditions matter Am J Obstet Gynecol 2016 215 05 583 5870 10.1016/j.ajog.2016.06.045 27793311

